# Case of Extra-Uterine Conception, with the Appearances on Dissection

**Published:** 1819-01-01

**Authors:** 

**Affiliations:** Physician to the Plymouth, Plymouth Dock, and Stonehouse Dispensaries


### *Case of Extra-Uterine Conception, with the Appearances on Dissection.*

By Dr. Cookworthy, Physician to the Plymouth, Plymouth Dock, and Stonehouse Dispensaries.

X. Y. thirty years of age, *belle et bien faite,* the wife of a husbandman, residing about a mile from this town, was admitted a patient of the Dock and Stonehouse Public Dispensary in the end of March last. She had a cough, with slight symptoms of fever, which yielded to bleeding, cathartics, and antimonials, the practice followed by Mr. Boyle, the apothecary in the above-mentioned Institution, who had the charge of her. On Monday the 13th of April she was reported convalescent.

On Thursday the 16th Mr. Boyle was again called. He found her with fever, and with pain and tension of the belly. He took ℥xij of blood from the arm, and gave some castor oil. On the following day (17th), the patient being thought dangerously ill, I was desired to see her for the first time. She was lying on her back in bed; her eye-brows were contracted; anxiety was depicted on her countenance; and the slowly altering motion of her limbs, satisfied me that some serious evil was at hand.

The patient, though perfectly alive to her situation, being a woman of strong mind, was not alarmed, neither did she complain unless when questioned. The heat of the skin, and the frequency of respiration were increased, and shivering was often mentioned. The artery at the wrist felt like a thread, and its pulsations were rapid. The tongue was white and dry. The bowels had been opened, and the discharges were offensive, and scybalous. The urine trickled from her as soon as it entered the bladder, and there had been for some hours a desire to make water, although a catheter passed in the morning proved the bladder to be empty. My attention was particularly directed to tbe belly, which was generally enlarged, tense, and exquisitely painful, if pressure were made below the ribs on the right side, as far forward as the pubis. Here, indeed, she always felt pain, although it was not at all times equally violent. There was also an uneasy feeling in the right thigh.

I directed a large opening to be made into one of the veins of the arm immediately, and permitted the blood to flow until the pain was said to have subsided, and the patient fainted. A large dose of calomel, followed by castor oil, brought away two chamber-basins full of lumpy and offensive *fæces*. The patient slept this night, but had many frightful dreams. She felt, however, much relieved in the morning, (18th). The expression of anxiety had disappeared, the belly was flaccid, and there was no pain in it, excepting that pressure shewed some tenderness above the right ilium. The tongue remained white and dry, and the apparent irritability of the bladder continued. An infusion of senna with jalap operated freely, and caused many fœtid and bilious discharges. I saw her again before night. She was quite cheerful, was grateful for the perfect relief afforded her, and asked for tea and biscuits. All the vital and animal functions seemed to be undisturbed; still the whiteness of the tongue, (although it was less dry) and the frequent micturition, convinced me that irritation existed somewhere; but not knowing where, nor of what nature it was, I resolved to wait until the disease might favour me with some more certain indication.

Whilst taking her tea, she informed me, that two years had passed since the birth of her last child, whom she had nursed up to the period of Mr. Boyle's attendance, at whose request he had been weaned: that she had menstruated regularly for several months; that she was in this state when her indisposition first assailed her, and that it had returned on the day when she was last bled. She was, therefore, sure she was not with child. Firmly believing that her present illness was connected with the first attack, which was thought to be catarrh, I questioned her respecting it. She acknowledged that she had had a cold, but thought the pain in her side was occasioned by carrying a heavy market basket to Plymouth on her right hip. She had not been well, nor free from pain since, and I found the circumstance had been made the subject of conversation in the family.

On the Sunday, 19th, she was nearly as on the preceding day. Enjoining quietness and perfect rest, spoon-diet, and the use of some castor oil; I quitted her in the same state of doubt and apprehension.

At seven o'clock on Monday morning, 20th, her husband came to me in great haste, saying, that his wife had slept composedly through the night, but soon after five the pains returned with great violence, and that they could only be likened to the pains of labour. He was a widower before he reached his home!

Twenty-four hours after death the body was examined, in the presence of Mr. Boyle and Mr. Welch, the apothecaries of the Dock Dispensary. Decomposition had scarcely begun. The belly was tumid, and evidently contained a fluid. There were no adhesions in the chest. The heart and lungs shewed no marks of disease. On dividing the peritonæum several points of bloody serum escaped, and more was afterwards removed. The stomach was healthy, and the liver was of a natural colour and size; but the gall-bladder was much enlarged, and contained a glairy fluid with seventy-nine calculi; one larger than the rest blocking up the cystic duct. The small intestines presented no unusual appearance, excepting that below the umbilicus they were glued together by coagulated blood. My curiosity was now excited. To see the real state of the parts, I continued the incision down to the pubis, and turned the parietes of the abdomen out over the thighs, and no sooner were the intestines raised, than a tumour, about the size of a large orange, was observed resting upon the right ilium, imbedded in blood. There was in its upper surface a laceration an inch and a half long, which, laying bare a transparent membrane, discovered the rudiments of a fœtus. I next carefully removed the blood which entirely filled the pelvis, and, by its compression, prevented the distension of the bladder. The bladder and kidnies were healthy; neither did the peritonæum, at any one part, appear inflamed. After searching a long while in vain for some ruptured vessel of consequence, from whence only, I thought, so much blood could so suddenly flow, I passed my knife close round the brim of the pelvis, and removed, *en masse,* its entire contents, the *rectum* only excepted. I was so fortunate as to bring them home without injury, and they now form an interesting preparation in the museum of my friend Mr. John Fuge, a surgeon in this town.

The uterus is rather large, and the neck elongated. The bloody jelly which occupied the mouth still adheres. There is no *membrana decidua.* Nothing like inflammation appears to have existed, except at the fundus (externally) where some coagulated blood adheres with unusual firmness. The left Fallopian tube, and the right ovarium are in a healthy state. The left ovarium contains a tumour, about the size of a nutmeg, which was full of a glairy fluid. The canal of the right Fallopian tube is closed throughout its whole extent, and its fimbriæ embrace the tumour above mentioned. The parietes of the tumour are made up of two substances, easily separable from each other, and of very different structure. The outermost layer is about half an inch thick, contains many patches of extravasated blood, and resembles the shaggy chorion. It is traversed by numerous blood-vessels, and several white threads which form the bond of union between it and the amnion within. Besides the laceration before spoken of, there is another very near the attachment of the Fallopian tube, where the vessels unite to form the *funis umbilicalis.* The fœtus is a male, perfect in all its parts, and about three inches long.

During the life-time of this patient, I believed her disease to be an inflammation of the peritonæum. I regarded the anxious expression of the face and body, the white and dry tongue, the small, hard, and rapid pulse, the tension and pain of the belly, and the frequent desire to make water, as certain signs of it. Still I was unable to account for the continuance of the white tongue and irritable bladder, after the lancet had removed the other less equivocal symptoms. The frequent micturition is, however, now explained. The dense coagulum which surrounded the bladder having, no doubt, prevented the collection of urine.

Of course, this woman died from hæmorrhage, proceeding from the lacerated parietes of the ovum ; and the dissection has taught me, (what perhaps I ought to have known before) that there may be much general irritation, great vascular action, even local pain, without inflammation. I am now disposed to think that, if the lancet had been more freely used, the ovum might have burst with less hæmorrhage, and the fœtus have found its way into some of the natural passages.

Conception, under such circumstances, is very justly termed *extra-uterine,* for the uterus can scarcely be said to have sympathized with it. The woman had menstruated for some months, and, supposing the left Fallopian tube and ovary sound, I see no reason why she might not have again conceived and brought forth a child. I do not mean to imply that the menstrual flux is *necessary* to impregnation; but, as I suppose it to proceed from the uterus, it was a proof in this case that the os tincæ was not sealed, notwithstanding the lymph found in the cervix.

*Frankfort Place, Plymouth,*

*August* 21, 1818.

